# Comprehensive analysis of the prognostic value and immune implications of the TTK gene in lung adenocarcinoma: a meta-analysis and bioinformatics analysis

**DOI:** 10.1080/19768354.2022.2079718

**Published:** 2022-05-24

**Authors:** Bo Li, Xiaojuan Gu, Hanbing Zhang, Hao Xiong

**Affiliations:** Department of Respiratory and Critical Care Medicine, The Second People's Hospital of Yibin, Yibin City, People’s Republic of China

**Keywords:** TTK, prognosis, meta-analysis, lung adenocarcinoma, TCGA

## Abstract

**Background:**

High expression levels of the TTK gene are closely related to tumor occurrence and poor prognosis, as confirmed by some studies. Our study explored the prognosis of lung adenocarcinoma (LUAD) patients with different TTK levels and the possible pathological mechanism of TTK in LUAD.

**Methods:**

We extensively searched literature databases and high-throughput sequencing databases and included relevant literature or datasets in the meta-analysis according to the inclusion and exclusion criteria. Hazard ratios (HRs) and 95% confidence intervals (CIs) related to TTK expression were calculated, publication bias was assessed, and sensitivity tests were performed. We also compared the relationship between cancer immune infiltrating cells and tumor mutation burden (TMB) in patients with different TTK expression levels via bioinformatics analysis. The half maximal inhibitory concentration (IC50) of some chemotherapeutic and targeted therapy drugs were calculated. The potential biological functions or pathways associated with different TTK expression levels were determined by gene set enrichment analysis (GSEA).

**Results:**

The meta-analysis revealed that higher TTK expression level was significantly associated with poor prognosis in LUAD patients, both in overall survival (OS) and progression-free survival (PFS). The expression level of TTK was significantly correlated with presence of some immune cells and TMB. Tumors with higher TTK expression levels were mostly enriched for the cell cycle, DNA replication and homologous recombination pathways. In addition, patients with different TTK expression levels were differently sensitive to some antitumor drugs.

**Conclusion:**

TTK may be a promising prognostic biomarker for LUAD and is worthy of further investigation.

## 1. Introduction

The latest global cancer epidemiological survey from 2021 suggests that 1,898,160 patients will receive a cancer diagnosis in the United States and that 608,570 people will die from cancer. Among cancers, lung cancer is the leading cause of cancer death in men and women. Lung cancer in patients without a history of smoking should not be underestimated and is expected to cause 20,420 deaths (Siegel et al. 2021). In addition, the latest Chinese tumor registration data show that there were approximately 3.929 million new cases of malignant tumors and approximately 2.338 million deaths from malignant tumors in China. Among all malignant tumors, lung cancer ranks first in incidence and mortality (Feng et al. 2019). According to cancer epidemiological statistics from around the world, cases of lung cancer and deaths from lung cancer in China account for 37.0% and 39.8% of those in the world, respectively (Sung et al. 2021). Lung cancer is usually divided into nonsmall-cell lung cancer (NSCLC) and small-cell lung cancer (SCLC). Among these, NSCLC accounts for the vast majority of cases, and the most common pathological type of NSCLC is lung adenocarcinoma (LUAD).

Chromosome segregation errors will lead to inaccurate distribution of genetic material to daughter cells during cell division and to defects such as chromosome rearrangement, loss of specific genomic regions or loss of whole chromosomes. These defects may lead to cell death or tumor development (Rutledge and Cimini 2016). TTK is a dual-targeted specific protein kinase that can phosphorylate serines/threonines and tyrosines and is the core component of the spindle assembly checkpoint (SAC). The SAC is a key monitoring mechanism that prevents inappropriate chromosome division by delaying the process of mitosis until all chromosomes are correctly attached to spindle microtubules, which can ensure the accurate separation of chromatids (Liu and Winey 2012). A recent review reported that high TTK expression is commonly found in many types of human malignant tumors (Xie et al. 2017).

The wide promotion of precision medicine and the continuous development of next-generation sequencing technology have enabled the emergence of gene- and molecule-targeted precision therapies, which are promising strategies in the clinic (van Dijk et al. 2014; König et al. 2017). Targeted therapy based on patient genetic sequencing results has shown good therapeutic efficacy in clinical cases. Therefore, it is necessary to explore better therapeutic targets and improve treatment. In view of the key function of TTK in cell division, it may become a new therapeutic target and biomarker of LUAD. Therefore, in this study, we explored the effect of TTK on the prognosis of patients with LUAD by performing a meta-analysis of all relevant studies. In addition, we conducted an analysis of a vast amount of data to explore the effect of TTK on the efficacy of some common drug treatment strategies used in LUAD patients and its relationship with immune infiltration.

## 2. Patients and methods

### 2.1. Database search

PubMed, EMBASE, Web of Science and Cochrane Library were systematically searched by two researchers. The search terms were set as hMps1, TTK, MPS1L1, ESK, Mps1, PYT and lung adenocarcinomas, lung adenocarcinoma, adenocarcinoma, lung and adenocarcinomas, and lung. Boolean logic operations were conducted according to the different literature databases. Inclusion criteria included (1) study type: prospective or retrospective cohort study; (2) participants: patients with pathologically diagnosed LUAD expression of TTK in tumor tissues; and (3) outcome data: overall survival (OS), progression-free survival (PFS), and OS and PFS hazard ratio (HR) and 95% CI data, or sufficient data to enable calculation of these outcomes. Literature exclusion criteria included (1) nonoriginal studies such as reviews, conference summaries and case reports; and (2) animal experiments or cell experiments. If multiple studies with the same content were identified, the most recent and most complete study was used. In addition, we also searched two public databases, The Cancer Genome Atlas (TCGA) and the Gene Expression Omnibus (GEO) database, for sequencing data and clinical information. These two databases were employed to calculate the expression of TTK and its relationship with the OS and PFS of LUAD patients.

### 2.2. Data extraction

Two researchers searched the literature independently, screened the literature according to the inclusion and exclusion criteria, and extracted the data from the screened literature. The main data extracted were the HR and 95% CI values related to OS and PFS. If there were different opinions, they was discussed by the two researchers, or a third researcher made the decision. The limma (Ritchie et al. 2015) package was used to standardize and log2 transform the TCGA and GEO data. In addition, we performed Cox regression analysis of the TTK expression and corresponding prognostic data to calculate the HR and 95% CI values.

### 2.3. Quality assessment

The literature quality was evaluated by the Newcastle Ottawa Scale (NOS). The NOS scoring system includes three aspects: study object selection, intergroup comparability and result measurement, for a total of 8 items (Cook and Reed 2015). The maximum score for each item in the study object selection and result measurement categories was 1 point, and the maximum score for each item in the intergroup comparability category was 2 points. The NOS score ranges from 0 to 9. The higher the score, the higher the quality of the included literature. A score of more than 6 indicates that the quality of the literature is good. The above process was carried out independently by two researchers. If there was a difference, it was discussed by the two researchers, or a third researcher made the decision.

### 2.4. Meta-analysis

In this study, Stata 15.0 software was used for analysis, and HR and 95% CI values were calculated. Heterogeneity was tested with Cochran's Q (Ng et al. 2008) and Higgins I2 (Higgins et al. 2003) tests. When *p* < 0.05 and I2 ≥ 50%, the heterogeneity was considered large, and a random effects model was used. When *p* > 0.05 and I2 < 50%, the heterogeneity was considered small, and a fixed effects model was used. The bias of the included literature was evaluated by Egger's funnel test. *P* < 0.05 was considered to indicate a statistically significant difference and the presence of publication bias. Finally, we conducted a sensitivity test by excluding each study one by one.

### 2.5. Construction of the nomogram

Because the TCGA database has the most detailed clinical information and sequencing data of patients, we used TCGA data to establish a prognostic nomogram related to TTK expression, and the subsequent analysis also comes from the data of TCGA database. The median value of TTK expression was used to divide patients into high and low expression groups. Cox regression analysis was used to determine independent prognostic factors. We then used the rms package to generate a nomogram based on these prognostic factors. Time-dependent ROC curves (Kamarudin et al. 2017) and calibration curves (Lindhiem et al. 2020) were used to assess the prediction ability of the nomogram.

### 2.6. Estimation of immune infiltration levels

CIBERSORT (https://cibersort.stanford.edu) is an online analysis tool for estimating gene expression profiles and estimating the percentage of cell types in mixed cell populations using gene expression data (Newman et al. 2015). First, we uploaded the mRNA data to the CIBERSORT database, and then, we used online analysis tools to set the number of permutations to 1000 and obtained the percentages of 22 kinds of immune cells in each sample. Finally, the proportions of various immune cells between the high and low TTK expression groups were compared.

### 2.7. Calculation of the tumor mutation burden (TMB) and processing of the somatic alteration data

The VarScan2 pipeline somatic mutation calling workflow was used (Binatti et al. 2021), and the read.maf function was used to read the somatic variants of each sample via the maftools package. The TMB value of each sample was calculated with the Perl script based on the Java8 platform and expressed as the total number of somatic mutations/sequencing region size (in mutations/Mb).

### 2.8. Exploration of the significance of the TTK expression level in clinical treatment

To evaluate the significance of the TTK expression level in clinical LUAD treatment, we calculated the half maximal inhibitory concentration (IC50) of common chemotherapeutic and targeted therapy drugs in the Cancer Genome Project (CGP) data of the LUAD dataset via the pRRophetic package (Geeleher et al. 2014).

### 2.9. Gene set enrichment analysis (GSEA)

C5.go.bp.v7.4.symbols.gmt, c5.go.cc.v7.4.symbols.gmt, c5.go.mf.v7.4.symbols.gmt and c2.cp.kegg.v7.4.symbols.gmt were selected as reference gene sets for the GSEA. The number of random permutations for each analysis was set to 1000. The level of TTK expression was used as a marker. The relevant signal channels enriched in each watch were sorted by *P* value and normalized enrichment score. In this study, the gene sets were analyzed by channel enrichment, the screening standard was set as FDR < 0.05, and the analysis results were visualized with the ggplot2 package.

### 2.10. Statistical analysis

The statistical calculations and analyses involved in this study were completed with R version 3.6.0 and stataSE 15.0. The Wilcoxon test was used to compare the differences between the two groups of variables. The patients were divided into high and low TTK expression groups according to the median value. Kaplan−Meier curves were drawn, and the differences were compared by log-rank analysis. OS was defined as the time from randomization to death from any cause. PFS was defined as the time from randomization to tumor progression or death from any cause. The correlation between two continuous variables was analyzed by Spearman correlation analysis. *P* < 0.05 was considered to indicate statistical significance.

## 3. Results

### 3.1. Literature and datasets search

Through extensive searches of the four literature databases according to the inclusion and exclusion criteria, we found that most studies were based on big data analysis obtained from public sequencing databases such as TCGA. Therefore, the data used in our analysis came from TCGA and GEO databases. Seventeen datasets, including GSE3141 (Bild et al. 2006), GSE8894 (Lee et al. 2008), GSE13213 (Tomida et al. 2009), GSE14814 (Zhu et al. 2010), GSE19188 (Hou et al. 2010), GSE26939 (Wilkerson et al. 2012), GSE29013 (Xie et al. 2011), GSE30219 (Rousseaux et al. 2013), GSE31210 (Okayama et al. 2012), GSE37745 (Lohr et al. 2015), GSE41271 (Girard et al. 2016), GSE42127 (Tang et al. 2013), GSE50081 (Der et al. 2014), GSE68465 (Shedden et al. 2008), GSE72094 (Schabath et al. 2016), GSE83227 (Bhattacharjee et al. 2001) and TCGA including 2886 LUAD patients were included in this meta-analysis. The included datasets were scored by NOS, which showed that all were high-quality studies. All included features are shown in Table 1.

**Table 1.** The clinical characteristics and NOS score of included datasets.

Datasets	First author	Year	Platform	Country	PMID	NOS score
GSE3141^15^	Andrea H Bild	2005	GPL570 [HG-U133_Plus_2] Affymetrix Human Genome U133 Plus 2.0 Array	USA	16273092	7
GSE8894^16^	Eung-Sirk Lee	2007	GPL570 [HG-U133_Plus_2] Affymetrix Human Genome U133 Plus 2.0 Array	South Korea	19010856	8
GSE13213^17^	Shuta Tomida	2008	GPL6480 Agilent-014850 Whole Human Genome Microarray 4×44K G4112F (Probe Name version)	Japan	19414676	9
GSE14814^18^	Chang-Qi Zhu	2009	GPL96 [HG-U133A] Affymetrix Human Genome U133A Array	Canada	20823422	8
GSE19188^19^	Jun Hou	2009	GPL570 [HG-U133_Plus_2] Affymetrix Human Genome U133 Plus 2.0 Array	Netherlands	20421987	8
GSE26939^20^	Wilkerson MD	2011	GPL9053 Agilent-UNC-custom-4X44K	USA	22590557	8
GSE29013^21^	Yang Xie	2011	GPL570 [HG-U133_Plus_2] Affymetrix Human Genome U133 Plus 2.0 Array	USA	21742808	8
GSE30219^22^	Sophie Rousseaux	2011	GPL570 [HG-U133_Plus_2] Affymetrix Human Genome U133 Plus 2.0 Array	France	23698379	8
GSE31210^23^	Hirokazu Okayama	2011	GPL570 [HG-U133_Plus_2] Affymetrix Human Genome U133 Plus 2.0 Array	Japan	23028479	9
GSE37745^24^	Miriam Lohr	2012	GPL570 [HG-U133_Plus_2] Affymetrix Human Genome U133 Plus 2.0 Array	Sweden	26608184	8
GSE41271^25^	Luc Girard	2012	GPL6884 Illumina HumanWG-6 v3.0 expression beadchip	USA	27354471	8
GSE42127^26^	Hao Tang	2012	GPL6884 Illumina HumanWG-6 v3.0 expression beadchip	USA	23357979	9
GSE50081^27^	Sandy D Der	2013	GPL570 [HG-U133_Plus_2] Affymetrix Human Genome U133 Plus 2.0 Array	Canada	24305008	9
GSE68465^28^	Kerby Shedden	2015	GPL96 [HG-U133A] Affymetrix Human Genome U133A Array	USA	18641660	8
GSE72094^29^	M B Schabath	2015	GPL15048 Rosetta/Merck Human RSTA Custom Affymetrix 2.0 microarray [HuRSTA_2a520709.CDF]	USA	26477306	8
GSE83227^30^	A Bhattacharjee	2016	GPL8300 [HG_U95Av2] Affymetrix Human Genome U95 Version 2 Array	USA	11707567	8
TCGA^31^	Eric A Collisson	2020	Illumina Hiseq	USA	25079552	8

### 3.2. Meta-analysis

**Figure 1.**
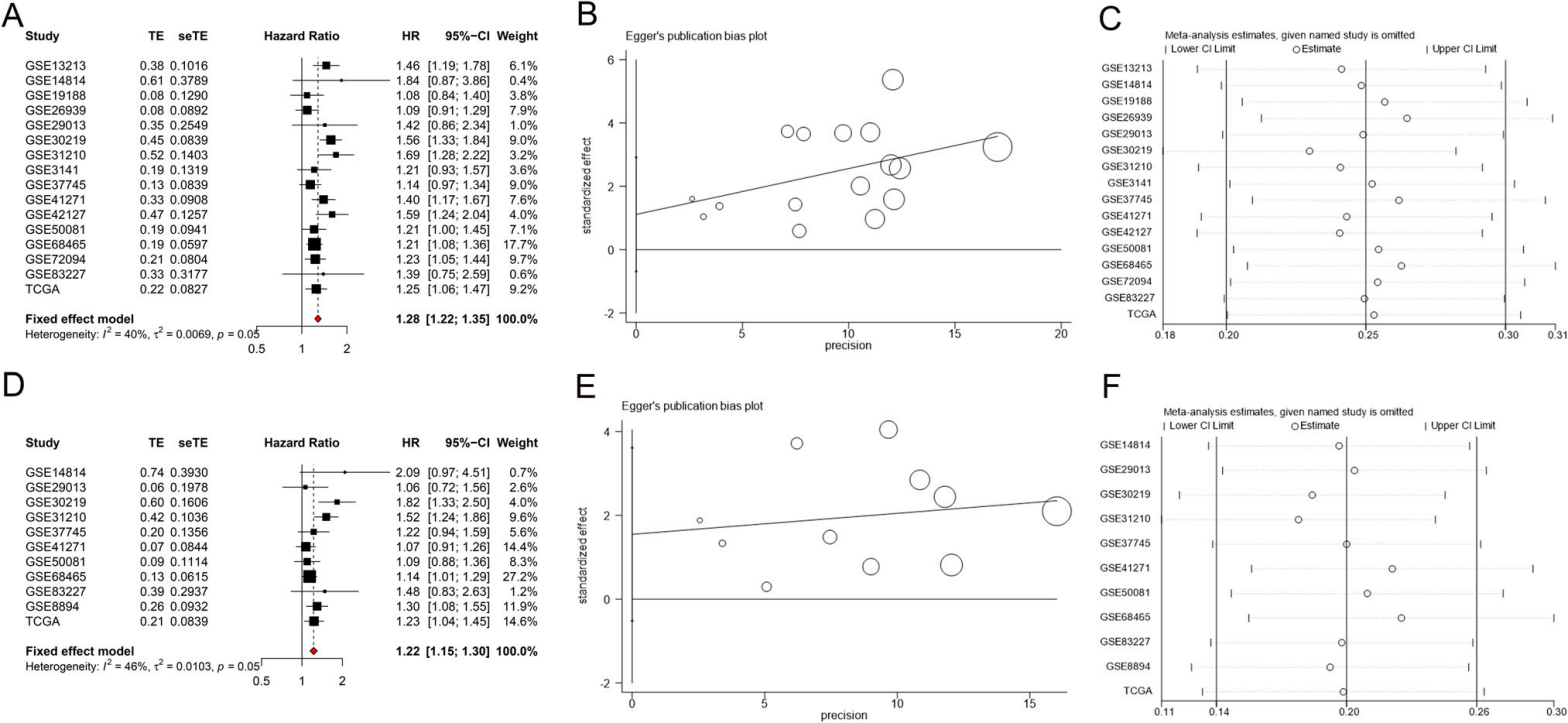
Meta-analysis results of TTK expression for overall survival (OS) (A) and progression-free survival (PFS) (D). Eggers test results of OS (B) and PFS (E). Sensitivity analysis results of OS (C) and PFS (F).

Sixteen datasets reported the relationship between TTK expression level and OS in LUAD patients. The heterogeneity among studies was I2 = 39.8%, *P* = 0.051, so the fixed effects model was used for analysis. The results showed that the OS of LUAD patients in the TTK high expression group was shorter (HR = 1.28, 95% CI 1.22∼1.35, *P* < 0.01, Figure 1A), and the difference was statistically significant. Eleven datasets reported the relationship between TTK expression level and PFS in LUAD patients. The heterogeneity among studies was I2 = 46.0%, *P* = 0.050, so the fixed effects model was used for analysis. The results showed that the PFS of LUAD patients in the TTK high expression group was also shorter (HR = 1.22, 95% CI 1.15∼1.30, *P* < 0.01, Figure 1D), and the difference was statistically significant. Egger's test suggested that there was no publication bias in the analysis of OS, with a *P* value of 0.208 (Figure 1B), and in the analysis of PFS, with a *P* value of 0.123 (Figure 1E). Sensitivity analysis suggested that excluding relevant studies one by one had little effect on the overall results of OS (Figure 1C) and PFS (Figure 1F).

### 3.3. Construction of the nomogram

**Figure 2.**
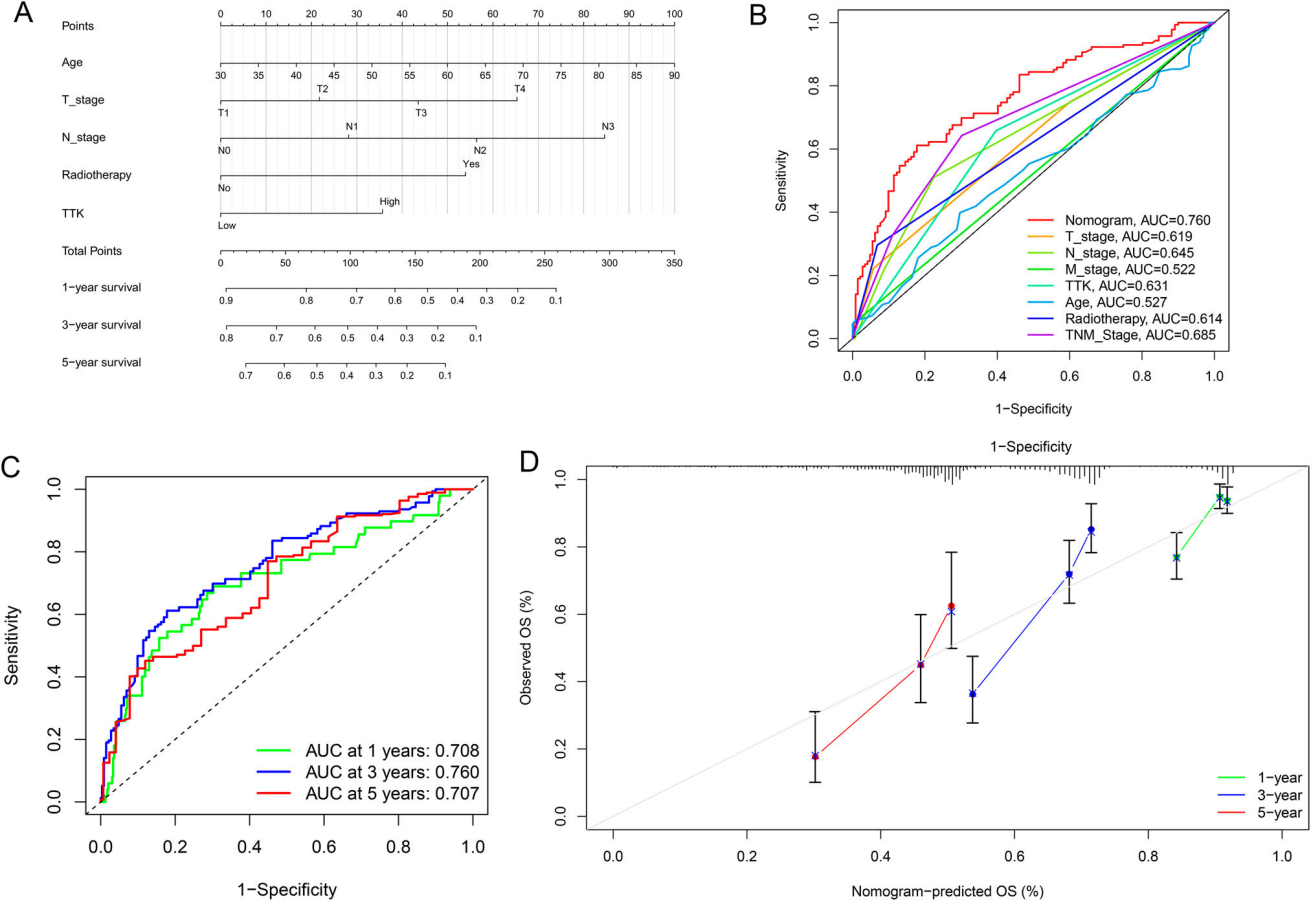
(A). A nomogram containing TTK expression level. (B). The receiver operating characteristic (ROC) curve contained TTK expression and TNM stage. (C). Time dependent ROC curve. (D). Calibration curve for the nomogram.

Multivariate Cox regression analysis revealed that age, T stage, N stage, radiotherapy and TTK expression level were independent prognostic factors (Table 2). The nomogram is shown in Figure 2A. The nomogram had better prediction ability than those of age, T stage, N stage, M stage, TNM stage, radiotherapy and TTK expression level, with an area under the curve (AUC) of 0.760 (Figure 2B). Moreover, the AUCs of the nomogram at 1, 3 and 5 years were 0.708, 0.760 and 0.707, respectively (Figure 2C). The calibration curve suggested that the nomogram had good prediction ability (Figure 2D).

**Table 2.** Results of the multivariate Cox regression analysis of TCGA cohort.

id	coef	HR	HR.95L	HR.95H	pvalue
Age	0.020	1.020	1.003	1.038	0.022
Sex	−0.033	0.967	0.695	1.346	0.843
Race	0.028	1.028	0.690	1.533	0.892
T stage	0.248	1.281	1.043	1.574	0.018
N stage	0.408	1.503	1.225	1.845	0.000
M stage	0.430	1.538	0.777	3.042	0.217
chemotherapy	−0.357	0.700	0.480	1.021	0.064
Radiotherapy	0.730	2.074	1.400	3.074	0.000
Smoking history	−0.107	0.898	0.559	1.443	0.658
TTK expression	0.462	1.587	1.129	2.232	0.008

### 3.4. Estimation of immune infiltration levels

**Figure 3.**
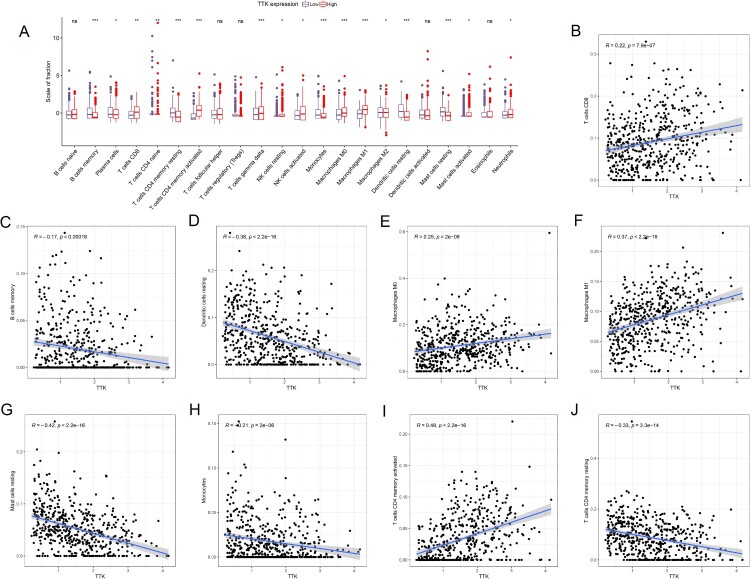
(A). The degree of 22 tumor immune-infiltrating cells in patients with high and low TTK expression. Scatterplots depicting the correlation between TTK expression and (B) CD8 T cells, (C) memory B cells, (D) resting dendritic cells, (E) M0 macrophages, (F) M1 macrophages, (G) resting mast cells, (H) monocytes, (I) activated memory CD4 T cells, and (J) T cells CD4 memory resting.

The percentages of 22 immune cells in LUAD patients from TCGA database were obtained by the CIBESORT algorithm. As shown in Figure 3A, we found that patients with a high TTK expression level had higher T cells CD8, T cells CD4 naive, T cells CD4 memory activated, T cells gamma delta, NK cells resting, NK cells activated, Macrophages M0, Macrophages M1, Mast cells activated and Neutrophils; a low TTK expression level was associated with higher B cells memory, Plasma cells, T cells CD4 memory resting, Monocytes, Macrophages M2, Dendritic cells resting and Mast cells resting. There was no significant difference in naive B cells, follicular helper T cells, regulatory T cells (Tregs), activated dendritic cells or eosinophils between high and low TTK patients. The expression level of TTK in LUAD patients was positively correlated with CD8 T cells (Figure 3B), M0 macrophages (Figure 3E), M1 macrophages (Figure 3F) and activated memory CD4 T cells (Figure 3I). The expression level of TTK in LUAD patients was negatively correlated with memory B cells (Figure 3C), resting dendritic cells (Figure 3D), resting mast cells (Figure 3G), monocytes (Figure 3H), and resting memory CD4 T cells (Figure 3J).

### 3.5. Processing of tumor mutation burden (TMB) and somatic alteration data

**Figure 4.**
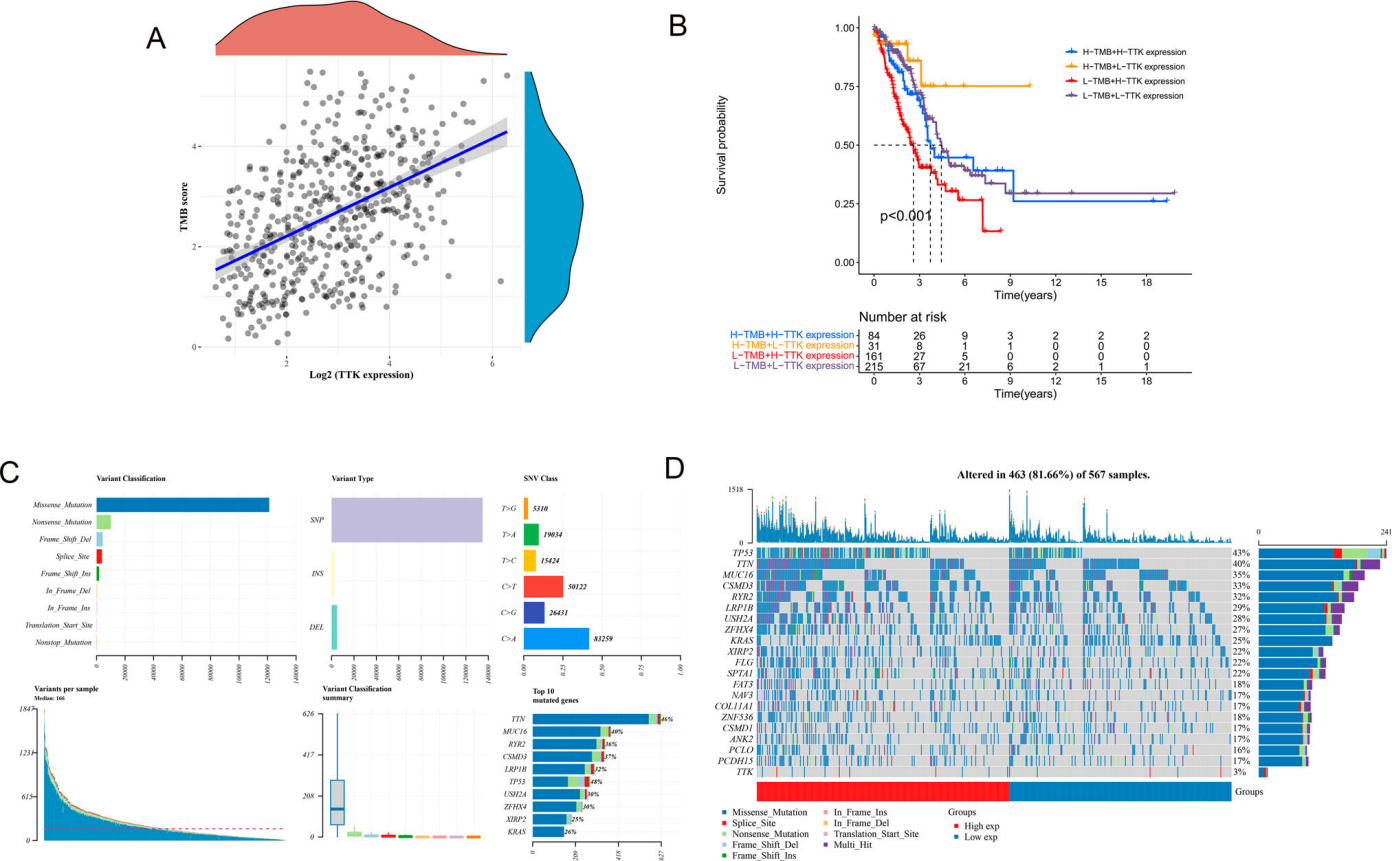
(A). Scatterplots depicting the correlation between TTK expression and tumor mutation burden (TMB). (B). Kaplan–Meier curves for patients in the TCGA-LUAD cohort stratified by both TMB and TTK expression level. (C). Mutation types, single nucleotide variation classification, single sample mutation variables and genes with the top 10 mutation frequencies in LUAD patients. (D). The oncoPrint was constructed using high and low TTK expression levels. Individual patients are represented in each column.

There was a significant positive correlation between TTK expression and TMB (Figure 4A). Moreover, when TMB was used as a stratification factor, the prognosis of patients with high TTK expression was significantly worse than that of patients with low TTK expression (Figure 4B). The mutation frequency of LUAD patients was very high, among which missense mutations, nonsense mutations, single nucleotide mutations (SNPs) and C>A mutations were more frequent (Figure 4C). The top 10 mutated genes were TTN, MUC16, RYR2, CSMD3, LRP1B, TP53, USH2A, ZFHX4, XIRP2 and KARS (Figure 4D).

### 3.6. Relationship between the expression level of TTK and antitumor therapy

**Figure 5.**
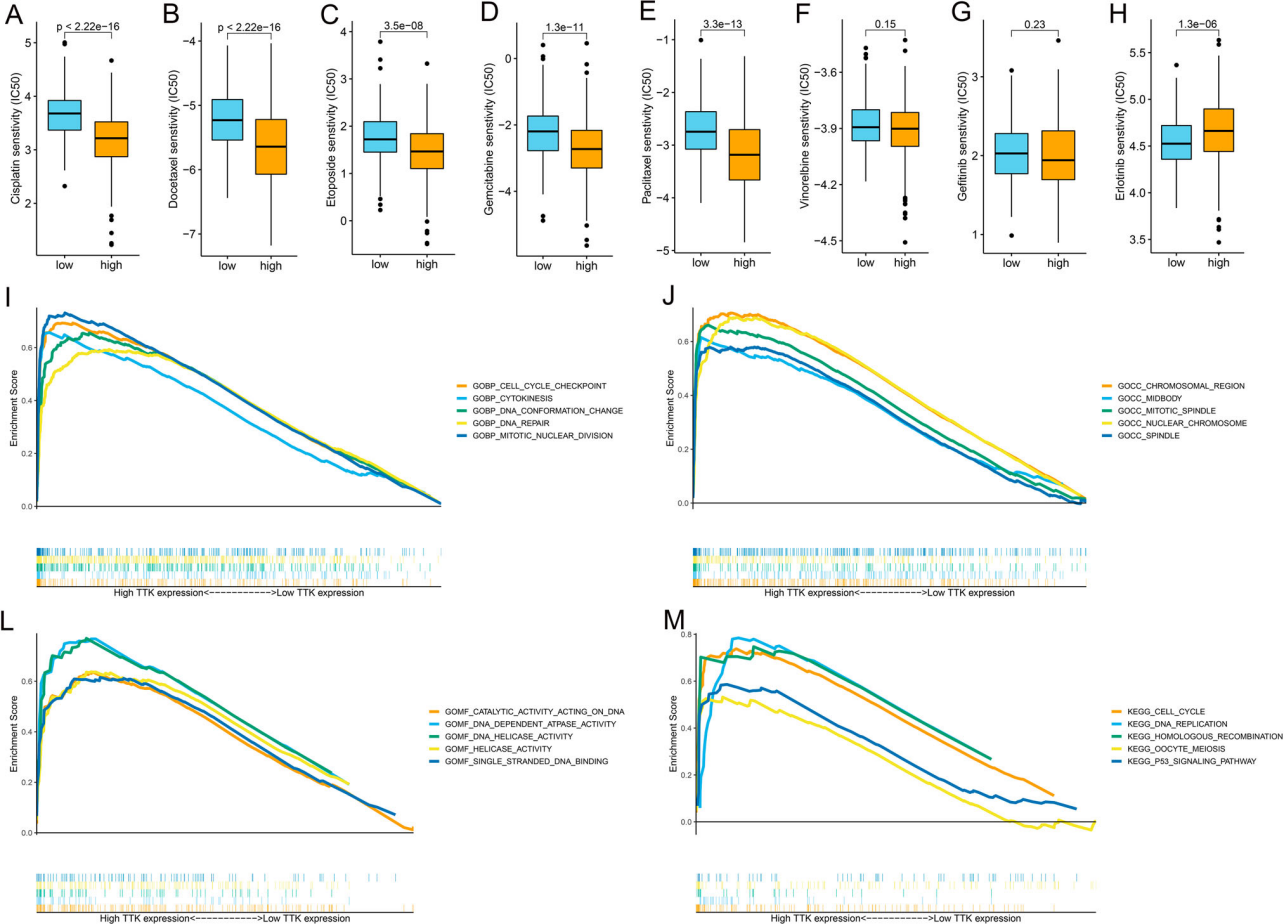
The relationships between different TTK expression levels and IC50 values for (A). Cisplatin, (B). Docetaxel, (C). Etoposide, (D). Gemcitabine, (E). Paclitaxel, (F). Vinorelbine, (G). Gefitinib and (H). Erlotinib. Enrichment plots showing the biological process (BP), cellular component (CC), molecular function (MF) and Kyoto Encyclopedia of Genes and Genomes (KEGG) in the high TTK expression group.

Several commonly used chemotherapeutic agents and targeted agents for LUAD were used to analyze the relationship between IC50 and high and low TTK expression levels in patients. Patients with higher TTK expression levels had lower IC50 values of cisplatin (Figure 5A), docetaxel (Figure 5B), etoposide (Figure 5C), gemcitabine (Figure 5D), and paclitaxel (Figure 5E), which means that these patients are more sensitive to treatment with these drugs. Patients with higher TTK expression levels had a higher IC50 of erlotinib (Figure 5H), which means that these patients became more resistant to this drug. There was no significant difference in the IC50 of vinorelbine (Figure 5F) and gemcitabine (Figure 5G) or between patients with high and low TTK expression levels.

### 3.7. GSEA

GSEA was performed via Gene Ontology (GO) analysis and Kyoto Encyclopedia of Genes and Genomes (KEGG) analysis to reveal the potential biological functions or pathways associated with high levels of TTK expression. For biological process (BP), high TTK expression levels were mostly enriched in cell cycle checkpoints, cytokinesis and DNA repair (Figure 5I). For cellular component (CC), high TTK expression levels were mostly enriched in chromosomal regions, midbodies and mitotic spindles (Figure 5J). For molecular function (MF), high TTK expression levels were mostly enriched in catalytic activity acting on DNA, DNA-dependent atpase activity and DNA helicase activity (Figure 5L). For KEGG, high TTK expression levels were mostly enriched in the cell cycle, DNA replication and homologous recombination (Figure 5L).

## 4. Discussion

In our study, we explored the influence of the TTK gene on the prognosis of LUAD patients, although we did not find any available literature after an extensive search of relevant literature databases. However, we obtained relevant data from TCGA and GEO databases for individual patient data (IPD) meta-analysis and found that high expression of TTK leads to poor prognosis. At the same time, we used clinical information in the TCGA database to construct a nomogram to prove the application of TTK in clinical prognosis. In addition, we also explored the relationships between the expression level of TTK and immune cell infiltration and antitumor management. The expression level of TTK is positively correlated with TMB, and the expression level of TTK is correlated with patient sensitivity or resistance to some antitumor drugs. Therefore, TTK is likely to become a LUAD tumor therapeutic target and prognostic marker.

The TTK gene, located on chromosome 6Q13-Q21, encodes serine/threonine and tyrosine protein kinases, which are doubly specific protein kinases (Liu and Winey 2012). TTK is the core component of SAC, which can ensure proper distribution of chromosomes to daughter cells, balance growth and division, and is a monitoring mechanism to ensure mitotic fidelity and genomic stability (Pachis and Kops 2018). When there is an abnormal connection between spindle microtubules and chromosomes, TTK monitors attach between centromeres and microtubules during metaphase, preventing metaphase to anmetaphase transition until all chromosomes are fully attached and develop on metaphase plates (Cuckle et al. 2015). In addition, Northern western blotting analysis showed that TTK transcripts were almost undetected in normal organs except the testis and placenta. However, high levels of TTK are readily detected in many types of human malignancies, and abnormal expression of TTK must affect SAC function (Xie et al. 2017).

At present, some studies have found that TTK may be related to the occurrence and development of lung cancer. Chen et al. found that USP9X expression increased in nonsmall-cell lung cancer. By using chemical markers, quantitative proteomic screening and other methods, USP9X RNA interference was analyzed in A549 lung cancer cells before and after, and functional experiments in vitro and in vivo were conducted. The results showed that TTK was a potential substrate of USP9X, and USP9X stabilized TTK through direct interaction and efficient deubiquitination of TTK on the K48 ubiquitin chain. When USP9X or TTK was knocked out, cell proliferation, migration and tumorigenesis were inhibited. Immunohistochemical analysis of NSCLC specimens showed that the protein expression levels of USP9X and TTK were significantly increased in tumor tissues (Chen et al. 2018). High levels of TTK in the tumor tissues of lung cancer patients promote the growth of cancer cells by upregulation of neurotensin, enhance the expression of DPYSL3, and promote cell migration and epithelial mesenchymal transformation, thereby enhancing the metastasis potential and ultimately leading to tumor metastasis, which is positively correlated with adverse clinical outcomes of patients (Tsai et al. 2020).

High TTK expression was found to be associated with later histological stage, higher TNM stage, lymph node metastasis, and poorer 5-year overall survival. According to the expression of TTK, Chen et al. divided 90 NSCLC patients into a low TTK expression group and a high TTK expression group, with 45 patients in each group. There was no significant difference in age, sex, location or distant metastasis between the low expression group and the high expression group. A high expression of TTK was significantly correlated with later histological stage and TNM stage of NSCLC. In addition, the incidence of lymph node metastasis in the high TTK expression group was significantly higher than that in the low TTK expression group. TTK expression was also correlated with the survival of patients, with an average 5-year overall survival of 49.7 months in the low expression group and 31.8 months in the high expression group (Chen et al. 2020). Zheng et al. showed that the expression of TTK was higher in lung adenocarcinoma and squamous cell carcinoma than in normal lung tissue, which was related to the poor prognosis of patients with lung adenocarcinoma (Zheng et al. 2019). Du et al. determined that LMO1 is an oncogene by combining the correlation between gene expression and patient survival rate and in vitro function. Its expression is related to the neuroendocrine differentiation of lung cancer, and it is a determinant of lung cancer invasiveness and prognosis, and TTK mediates the carcinogenic effect of LMO1 in lung cancer cells (Du et al. 2018).

The studies have shown that the expression level of some genes is related to poor tumor prognosis and immune infiltration of tumor cells (Alex and Alfredo 2020; Liao et al. 2021). In this study, we found that the expression level of TTK gene was significantly correlated with the immune infiltration level of B cells, T cells, macrophages, dendritic cells and with hypertrophy. B lymphocytes are immunized in the fluid of the tumor immune microenvironment to produce IgG antibodies and exert anti-tumor effects (Li et al. 2009). In addition, B cells can participate in tumor immunity as antigen-presenting cells. However, some studies have shown that regulatory B cells in the tumor microenvironment promote tumor growth and metastasis by secreting cytokines (Olkhanud et al. 2011). Therefore, B lymphocytes play a dual role in tumor immunity. As the most powerful antigen-presenting cells, dendritic cells can present antigens and provide costimulatory signals for the activation of T cells. At the same time, they can interact with NK cells and B cells to play an anti-tumor effect. The infiltration of mature and active dendritic cells in the tumor microenvironment increases immune activation and the recruitment of immune effector cells (Guillerey et al. 2016). The results of this study showed that the expression level of the TTK gene in LUAD was negatively correlated with the infiltration level of dendritic cells and B cells, indicating that TTK gene may lead to tumor progression and metastasis by promoting downregulation of the levels of these two immune cells in the immune microenvironment of LUAD.

Moreover, inhibitors targeting TTK have been shown to have antitumor effects. In a human cancer mouse model, oral administration of the TTK inhibitor cfi-402257 alone or in combination with an anti-programmed cell death 1 (PD-1) antibody inhibited tumor growth at a well-tolerated dose (Mason et al. 2017). Wu et al. found that the ATP binding cassette is a key factor leading to multidrug resistance, such as ABCG2. CC-671 is a strong selective inhibitor of TTK and CLK2. CC-671 is also an effective ABCG2 reversal agent. The reversal effect of CC-671 is mainly due to the inhibition of ABCG2 drug efflux activity, resulting in an increase in intracellular chemotherapeutic drug levels, which can improve the efficacy of chemotherapeutic drugs on ABCG2-overexpressing lung cancer cells (Wu et al. 2020). In addition, CC-671 did not change the protein expression or subcellular localization of ABCG2. Computational molecular docking analysis showed that CC-671 had high binding affinity with ABCG2 (Wu et al. 2020).

Our research still has some limitations. First, our meta-analysis is an IPD meta-analysis, not a conventional meta-analysis; our data were not limited to published data but we obtained the most original data, which greatly reduces the common publication bias and heterogeneity in conventional meta-analyses. However, our study was a retrospective analysis, and some confounding factors may not be corrected. Second, although we preliminarily explored the biological process of TTK in LUAD through GSEA, the detailed mechanism linking TTK expression with the pathological progress of LUAD needs to be verified by further biomedical experiments. However, the current results are encouraging and deserve attention in identifying promising predictive biomarkers of LUAD.

## 5. Conclusions

A high expression of TTK is related to poor prognosis in patients with LUAD. In addition, the expression level of TTK is correlated with some tumor infiltrating cells and is positively correlated with TMB. The expression of TTK in patients with LUAD may be related to the resistance to some antitumor drugs.

## Data Availability

The data generated within the study is shown in this manuscript. Any raw data or analysis is available from the corresponding author upon request.
